# Analysis of Replication Intermediates Indicates That *Drosophila melanogaster* Mitochondrial DNA Replicates by a Strand-Coupled Theta Mechanism

**DOI:** 10.1371/journal.pone.0053249

**Published:** 2013-01-04

**Authors:** Priit Jõers, Howard T. Jacobs

**Affiliations:** Institute of Biomedical Technology and Tampere University Hospital, University of Tampere, Tampere, Finland; Ben-Gurion University of the Negev, Israel

## Abstract

Mitochondrial DNA synthesis is necessary for the normal function of the organelle and for the eukaryotic organism as a whole. Here we demonstrate, using two-dimensional agarose gel electrophoresis to analyse replication intermediates, that unidirectional, strand-coupled DNA synthesis is the prevalent mode of mtDNA replication in *Drosophila melanogaster*. Commencing within the single, extended non-coding region (NCR), replication proceeds around the circular genome, manifesting an irregular rate of elongation, and pausing frequently in specific regions. Evidence for a limited contribution of strand-asynchronous DNA synthesis was found in a subset of mtDNA molecules, but confined to the ribosomal RNA gene region, just downstream of the NCR. Our findings imply that strand-coupled replication is widespread amongst metazoans, and should inform future research on mtDNA metabolism in *D. melanogaster*.

## Introduction

Faithful synthesis and propagation of the mitochondrial genome is an essential requirement for normal organellar and organismal function. Moreover, qualitative and quantitative mitochondrial DNA (mtDNA) aberrations underlie many human diseases (reviewed in [1]) and are widely considered to be a contributory factor in ageing [2]. Replication of the mitochondrial DNA (mtDNA) has therefore been a subject of intense study for more than 30 years.

Initial data based on transmission electron microscopy (TEM) of mammalian mtDNA purified by CsCl density-gradient centrifugation suggested a unidirectional, strand-asynchronous mode of replication (reviewed in [3]). According to this long-held view, replication initiates within the single, extensive non-coding region (NCR) of the genome, and proceeds unidirectionally by displacement-loop expansion. The synthesis of the second strand, using the displaced strand as a template, commences when the replication fork traverses a distinct origin approximately 2/3 of the way around the circular genome starting from the NCR. Initial TEM data from invertebrate model organisms was consistent with an analogous replication model, with strand-asynchronous replication intermediates reported in the sea urchin *Strongylocentrotus purpuratus* and the fruit fly *Drosophila melanogaster* [4], [5]. However, fully duplex Cairns-form, i.e. theta-type replicating molecules were also present in these organisms. Moreover, only theta-type *D. melanogaster* mtDNA replication intermediates (RIs) were reported in one earlier study [6].

The displacement-loop expansion model has subsequently been questioned by the use of two-dimensional neutral agarose gel electrophoresis (2DNAGE), a sensitive method widely employed for the analysis of replication intermediates (RIs). This method, developed almost three decades ago [7], and first used successfully to map replication origins and define the mode of replication of the 2μ plasmid in yeast [8], enables different types of non-linear DNA species to be resolved according to both their size and shape. After digestion with a restriction enzyme, RIs deriving from a given fragment, through which the replication machinery passes in a specific fashion, form an iterative series giving rise to characteristic arcs on 2DNAGE [9]. The method has been widely applied to map origins, characterize replication initiation mechanisms, and analyse fork stalling [10], [11], and its validity has been independently verified by TEM [12].

Applied to highly-purified mtDNA from vertebrates, this method demonstrated the presence of essentially double-stranded RIs, indicative of a strand-coupled mode of replication [13], [14]. However, these intermediates were reported to include a class containing extensive incorporation of RNA on the lagging strand [14]–[16]. Degradation of this RNA during isolation would produce partially replicated mtDNA molecules with large ssDNA regions, thus resembling intermediates predicted by the strand-asynchronous model. Indeed, partially single-stranded structures were observed using 2DNAGE when crude mtDNA preparations or those deliberately treated with ribonucleases were analyzed [15]. An alternate model has therefore been put forward, according to which replication proceeds in a strand-coupled manner, but with RNA incorporated throughout the lagging strand, as the replication fork advances. The debate about the mechanism of vertebrate mtDNA replication and the role of RNA therein is ongoing [16], [17].

Despite differences in gene order, the *Drosophila* mitochondrial genome closely resembles that of vertebrates in its overall structure and mode of organization [18]. Moreover, a similar set of enzymes and protein factors for DNA replication appears to be present in *Drosophila* as in vertebrate mitochondria, including DNA polymerase gamma, the T7 gp4-related helicase Twinkle (CG5924), and the mitochondrial single-stranded DNA binding protein mtSSB.

To address the continuing controversy over the mode of mtDNA replication, and taking account of the fact that two different classes of RI were inferred from previous TEM data in this organism [5], [6], [19], we set out to analyze *D. melanogaster* mtDNA replication by an independent method, i.e. 2DNAGE, starting from sucrose density gradient-purified mitochondria derived from cells and animals at different developmental stages. Our results confirm the previous inference of unidirectional, theta replication [5] initiating in the NCR [20]. Using 2DNAGE they also corroborate, but extend the earlier findings from TEM, showing two different types of RI, i.e. those that are fully double-stranded throughout their length, and a minority containing a region of single-strandedness on one branch. The use of 2DNAGE, in combination with a set of restriction enzyme digests extending over the entire mtDNA coding region, has enabled us to map the region of single-strandedness, that is seen in some molecules, to a short region adjacent to the NCR, encompassing the rRNA genes. This allows us to put forward a plausible mechanism accounting for the single-stranded region, i.e. delayed lagging-strand initiation in the heavily transcribed rRNA locus. Additionally, replication intermediates were inhomogeneously distributed around the genome, indicating systematic irregularities in the speed of replication fork progression, revealing specific pause regions.

## Results

### Replication is Unidirectional and Mainly Strand-coupled

**Figure 1:**
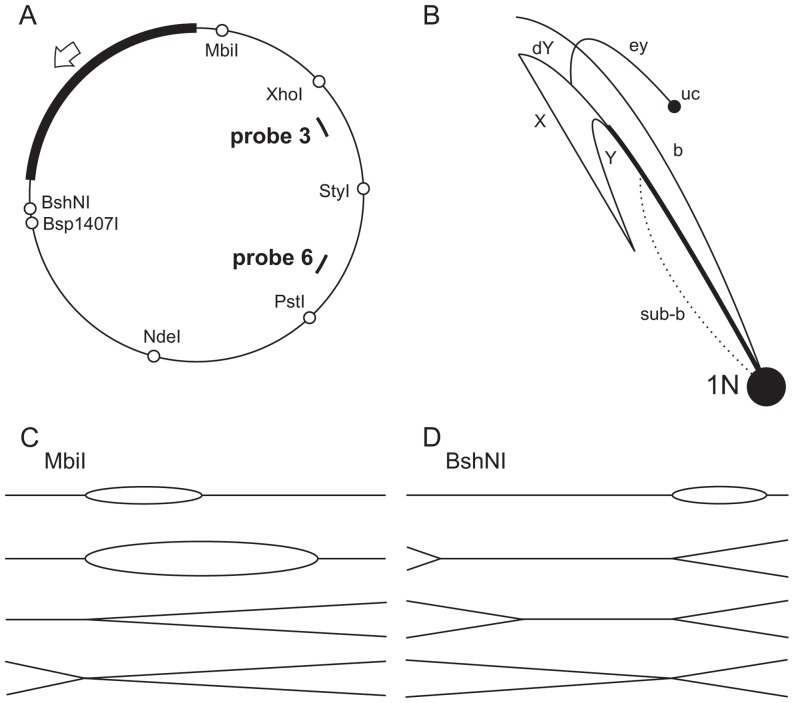
Schematic representation of the mitochondrial genome of *D. melanogaster*, showing major replication intermediates. A) Positions of unique restriction enzyme recognition sites in *D. melanogaster* mtDNA used in this study. Thick black line represents the NCR, arrow denotes the proposed direction of replication from the putative origin. Positions of probes used in this study are shown as bold lines. B) Diagram showing maximum spans of expected 2DNAGE migration arcs of major replication intermediates, following linearization of mtDNA with a generic restriction enzyme: b – bubble arc, Y – Y or Y-like arc (created by strand breakage of bubble or double Y species at the fork or origin), X – colliding forks and Holliday junctions, dY – double Y-arc, ey – “eyebrow” arc, sub-b – sub-bubble arc (bubble structures with ssDNA regions), uc – uncut circles, 1N - linearized non-replicating DNA (unit-length mitochondrial genome). Bold line extending from 1N spot marks the region where Y and dY structures migrate together. Schematic representation of expected intermediates generated by MbiI and BshNI digestions in case of unidirectional strand-coupled replication are shown in panels C and D, respectively.

*D. melanogaster* mitochondrial DNA was digested with restriction endonucleases having unique sites distributed around the genome (Figure 1A; see also Figure S1A in File SI), then analyzed by 2DNAGE. The method enables the identification of RIs according to their migration properties in agarose gels [9], [10], based on size and shape (Figure 1B; for more detailed description of RIs see Figure S1 in File SI). The initiation of standard, strand-coupled replication should produce molecules with an expanding replication bubble forming a characteristic slow-moving arc called the bubble arc (Figure 1B). The length of the bubble arc is indicative of how far the replication fork(s) proceeds in a circular molecule before encountering a point of cleavage by a given endonuclease [9], [10]. After the fork traverses this site, the bubble bursts, giving rise to a linear structure with branched ends (double-Y), since the replication fork immediately enters the linearized fragment from the other end (Figure 1B, dY; Figure 1C, D; Figure S1C in File SI). The length of a bubble arc in a series of overlapping restriction digests thus enables an origin to be mapped, and for its directionality to be ascertained [9], [10], [21].

**Figure 2:**
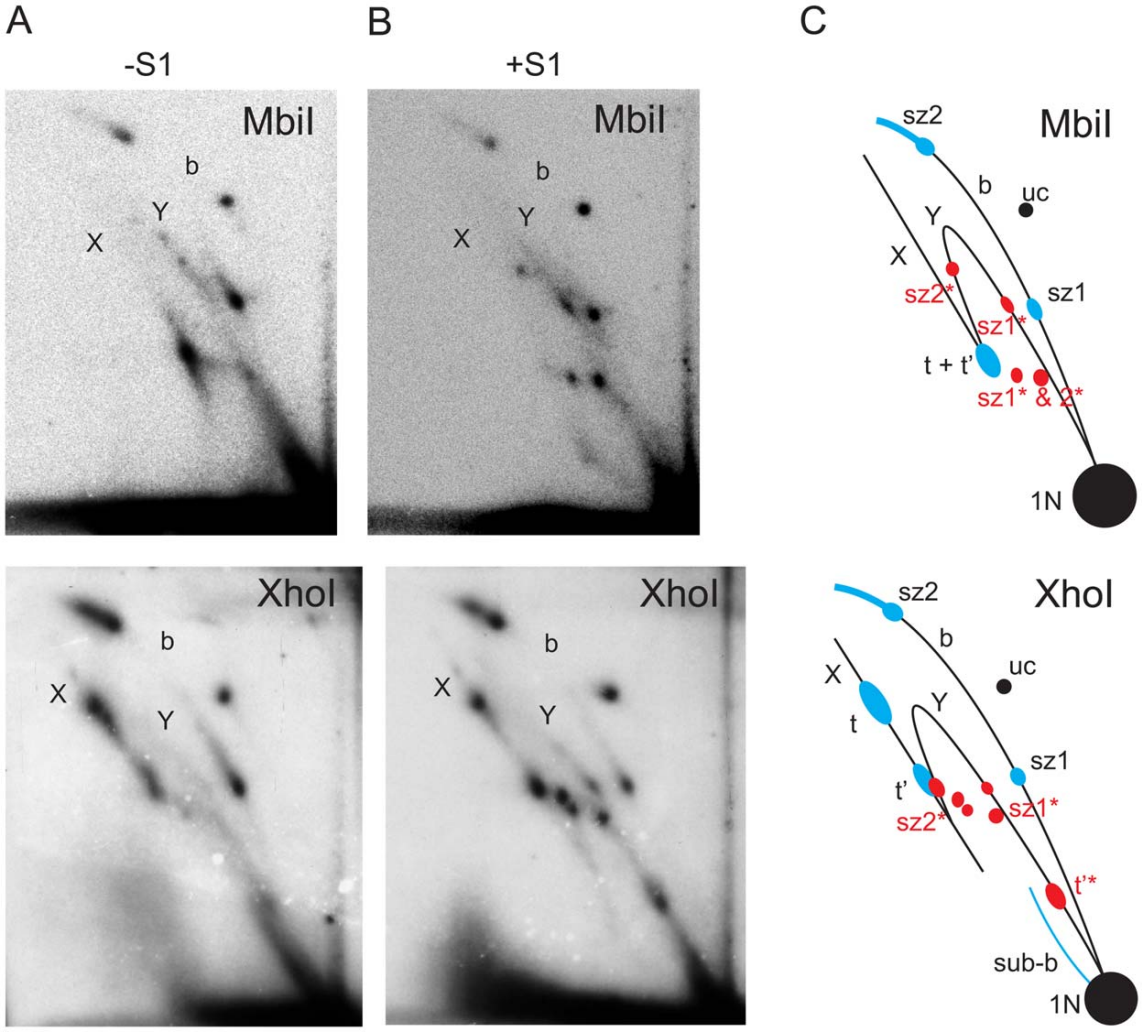
2DNAGE of MbiI- and XhoI-digested mtDNA from adult flies, with explanatory drawings. Replication intermediates from adult flies digested with MbiI and XhoI, hybridized with probe 6. Material was further digested with (panels B) or without (panels A) S1 nuclease. C) Summary drawing showing major replication intermediates inferred from the gels; t – fully duplex termination intermediates, t’ – termination intermediates with partially single-stranded character, sub-b – partially single-stranded bubble arc, sz1– replication slow-zone 1, sz2– replication slow-zone 2. Other forms are labeled as in Fig. 1. Species shown in blue are those most affected by S1 treatment; species shown in red represent new forms generated by S1 cleavage within regions having partially single-stranded character, either at the fork or at the origin. These are labeled with asterisks, based on the forms they derive from. Unlabeled species shown in red represent minor forms broken at the termination/origin/replication fork (see text). Note that the bubble arc signal between slow zones was only seen on long exposure of these gels. At long exposure, all arcs were continuous. The rather discrete segments of replication fork arcs seen on the shorter exposures, as shown, are consistent with unidirectional replication from a discrete origin, in cases where specific segments of the replicon are traversed at different rates.

**Figure 3:**
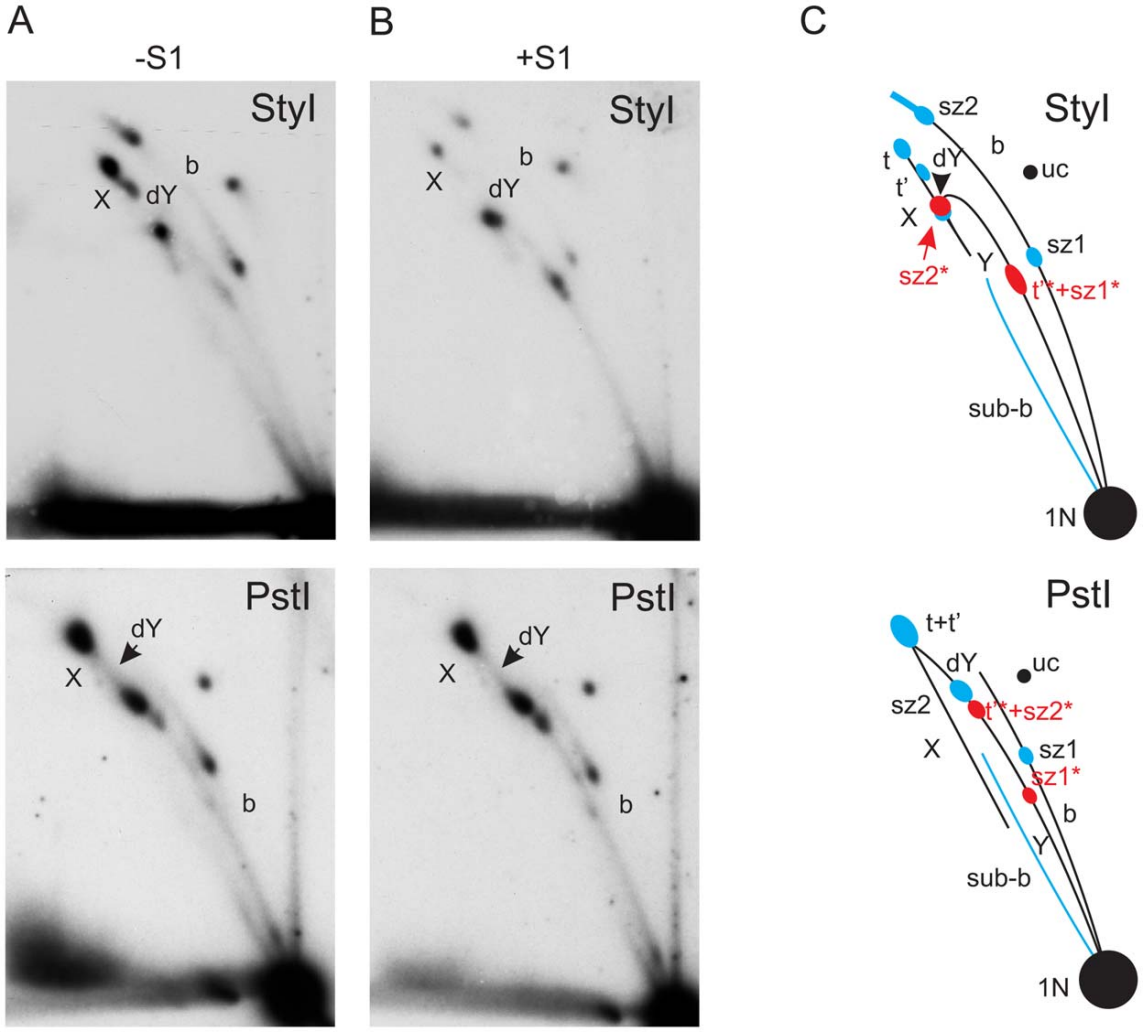
2DNAGE of StyI- and PstI-digested mtDNA from adult flies, with explanatory drawings. Replication intermediates from adult flies digested with StyI and PstI, hybridized with probe 6. Material was further digested with S1 nuclease (panels B) or was untreated (panels A). C) Summary drawing showing major replication intermediates inferred from the gels; species are labeled as in Figs. 1 and 2. Note the fusing of two termination intermediates into a single structure due to gel compression in the PstI digest and overlapping migration patterns of dY and sz2* in the StyI digest, for similar reasons.

**Figure 4:**
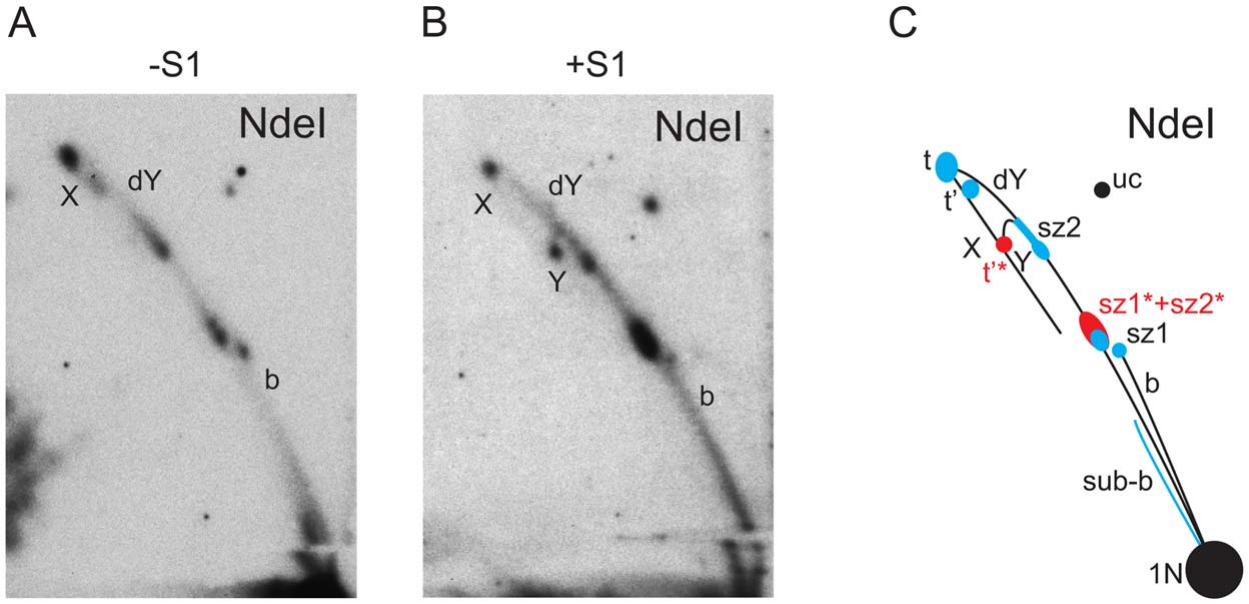
2DNAGE of NdeI-digested mtDNA from adult flies, with explanatory drawing. Replication intermediates from adult flies digested with NdeI, hybridized with probe 6. Material was further digested with S1 nuclease (panel B) or was untreated (panel A). C) Summary drawing showing major replication intermediates inferred from the gels; structures are labeled as in Figs. 1 and 2. Extensive 'slow region' following slow zone 2 but preceeding the final termination structure renders the apex of the Y-like arc visible after S1 nuclease treatment.

**Figure 5:**
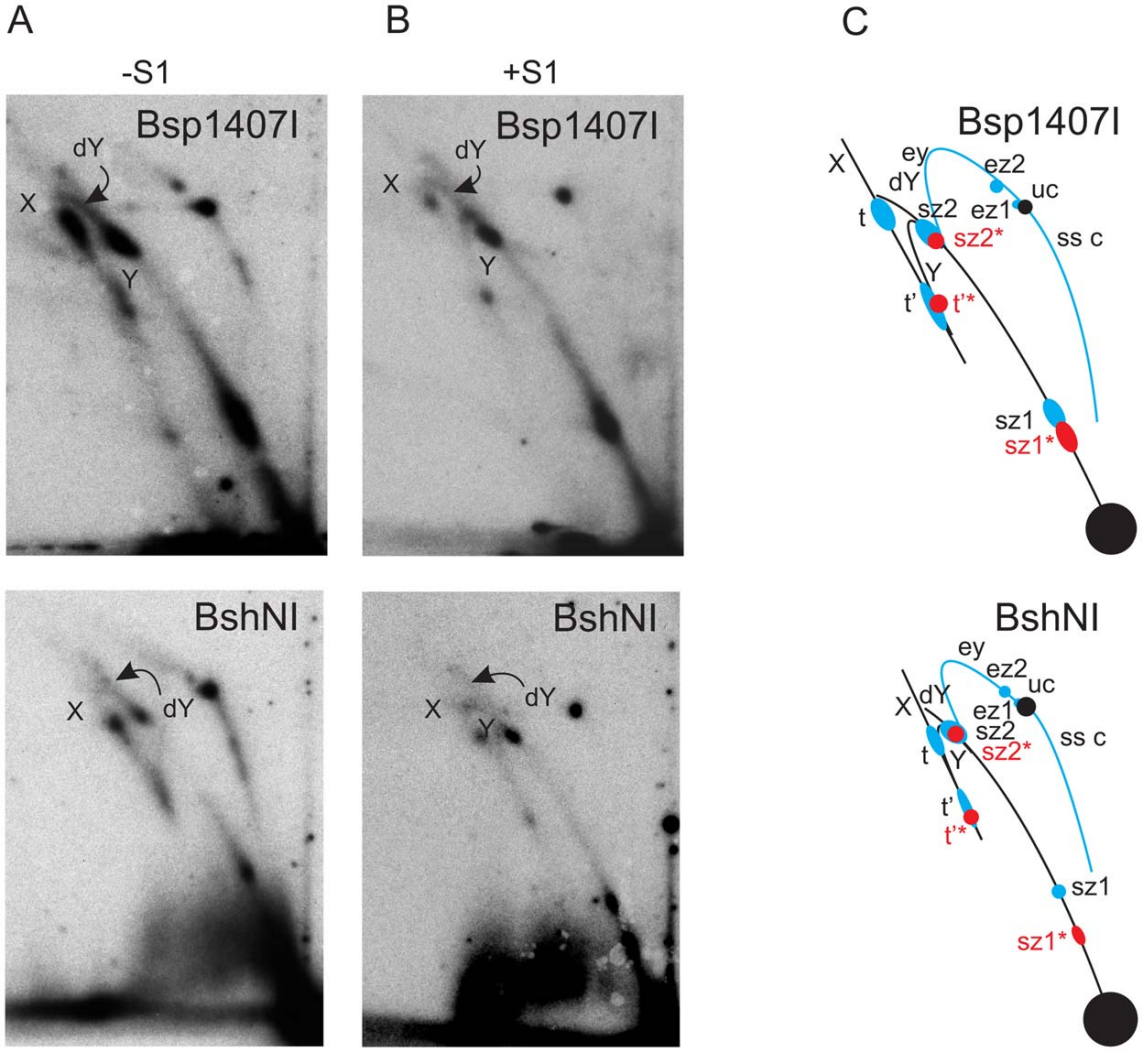
2DNAGE of Bsp1407I- and BshNI-digested mtDNA from adults, with explanatory drawings. Replication intermediates from adult flies digested with Bsp1407I and BshNI, hybridized with probe 6. Material was further digested with S1 nuclease (panels B) or was untreated (panels A). C) Summary drawing showing major replication intermediates inferred from the gels; ey – eyebrow arc, ss c – circles with variable ssDNA region, ez1 & 2– slow zones 1 & 2 on the eyebrow arc, other structures are labeled as in Figs 1 and 2. As in the case of the PstI digest, migration patterns of certain structures overlap before and after S1 treatment.

The maximum span of such a bubble arc was observed in material digested with MbiI, whose recognition site is adjacent to the NCR (Figures 1, 2N Figure S1 in File SI). The extent of the bubble arc was successively shortened, as the site of digestion was shifted around the genome from the MbiI site through the sites for XhoI to NdeI (Figures 2–4; Figure S1 in File SI). This is consistent with unidirectional replication starting within the NCR and entering the coding region at the rRNA locus, advancing in the same direction as that in which the rDNA is transcribed. See reference [21] for an example of the same reasoning applied to a bacterial plasmid. The apparent lack of any bubble arc signal in the Bsp1407I and BshNI digests (Figure 5) is not inconsistent with this hypothesis, since any short bubble arc would be impossible to resolve from the signal generated by the genome-length linear fragment (1n). A bubble arc of a discrete length, related to the positions of specific endonuclease cleavage sites, is also characteristic of a single major origin rather than a larger zone of random initiation [10]. Further support for this unidirectional, strand-coupled replication model comes from the observed reciprocal relationship between the length of the double-Y (dY) arc and that of the bubble arc. The length of the dY arc signal increased gradually as restriction sites successively closer to the rRNA locus were used, reaching its greatest length in the BshNI digest. This trend is seen most clearly when comparing the XhoI and Bsp1407I digests. In the former case there is hardly anything visible in the position of a dY arc whilst in the Bsp1407I digest a strong such arc with concentrations of material indicative of replication ‘slow zones’ can be seen to extend from near to the 1n spot. Note that, using this gel system, a dY arc will become clearly separable from a simple Y arc (equivalent also to a broken-bubble arc) only after the fork in simple Y structures has traversed beyond the centre of the fragment [9], whereupon the Y arc turns sharply downward (Figure S1B in File SI). Up until that point, Y (including broken bubble) and dY structures migrate very close together. Unfortunately, the region between the standard Y and X arcs where dYs will migrate is also very compressed, due to the gel-running conditions [22], [23] that must be employed to resolve fragments of the size of the linearized mitochondrial genome (15–20 kb). Multiple, less prominent intermediates are visible migrating below the Y arc after S1 nuclease treatment (e.g. Figure 2). They represent a collection of RIs in different elongation stages which we infer to be nicked by S1 at the origin/terminus/replication fork.

**Figure 6:**
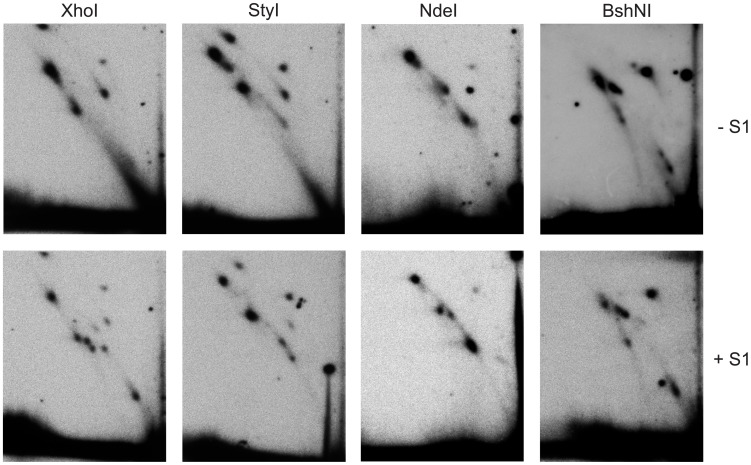
2DNAGE of mtDNA from Schneider S2 cell line. Replication intermediates from Schneider S2 cells, digested with the indicated enzymes, with or without subsequent S1 nuclease treatment, hybridized with probe 6. Migration paths are virtually identical with those of replication intermediates from *D. melanogaster* adults (compare with Figs. 2–5).

**Figure 7:**
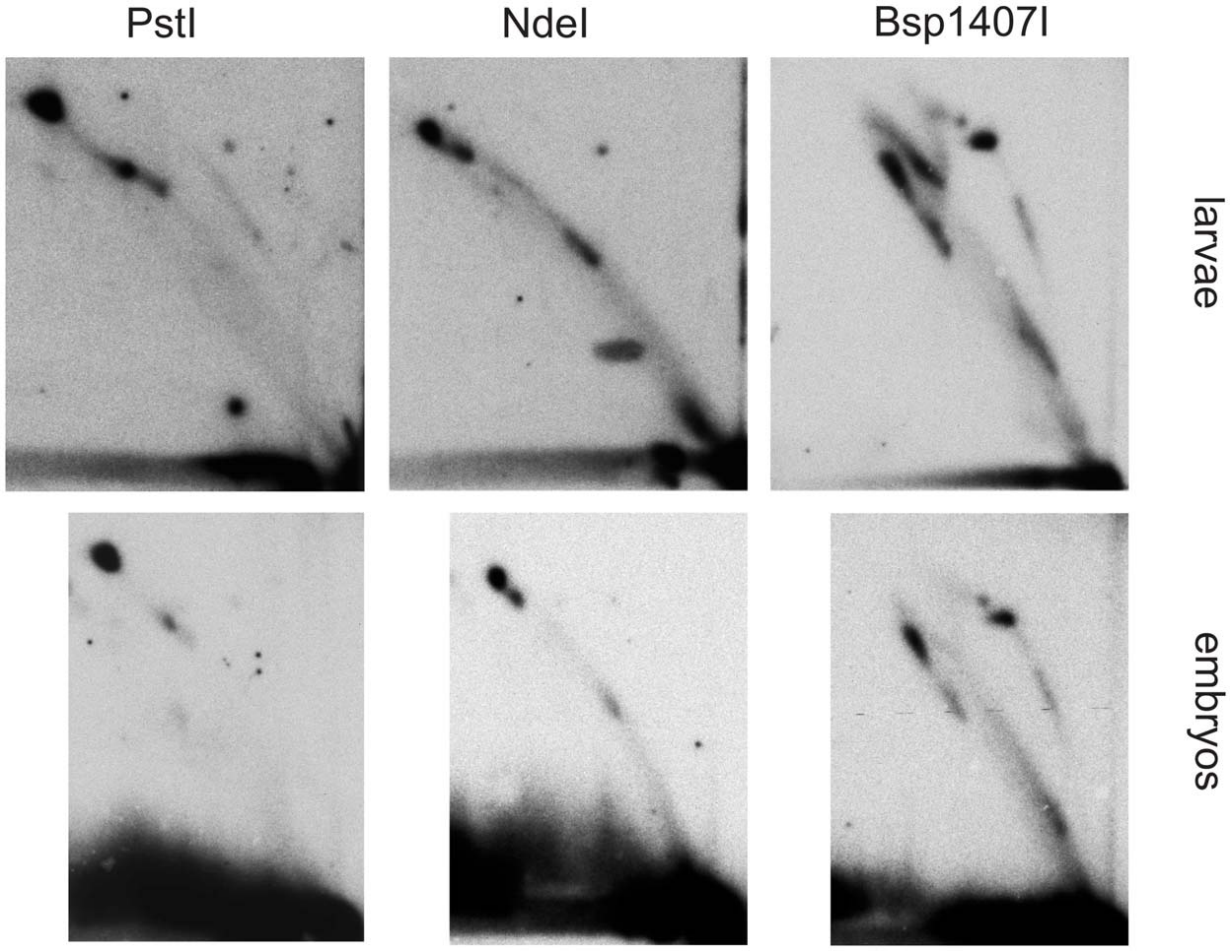
2DNAGE of mtDNA from larvae and embryos. Replication intermediates from *D. melanogaster* embryos and larvae, digested with the indicated enzymes and hybridized with probe 3. Note the rather prominent eyebrow on the Bsp1407I digest in larval mtDNA and the virtual absence of sz2 in replicating mtDNA from embryos.

Previous investigations by TEM, of the synthesis of mtDNA in *D. melanogaster* and in other *Drosophila* species, reported evidence for strand-asynchronous replication [5], [19]. Digestion of the intermediates predicted from this mode of replication by type II restriction endonucleases would result in cleavage of only one branch, since the displaced strand will remain single-stranded. The resulting products will comprise a persistent, covalently closed circle with increasing lengths of linear “arms” attached to it (Figures S1, S2, both in File SI). Such species would migrate on an eyebrow arc (for explanation see [24]), extending from uncut circles to the high molecular weight part of the gel (Figure 1B). In any case where both branches in such intermediates would be cut by the restriction enzyme, e.g. because it had activity against its target site in ssDNA, or due to the presence of just a short region of dsDNA around the site, the resulting products would have a lower molecular weight than fully double-stranded species, and their tendency to be retarded in the second-dimension gel would also be less. Thus, these structures would migrate below classical bubble, dY and Y arcs. However, with the exception of sites cutting in the rDNA region (see next section), the intermediates present in our 2D analysis have the mobility expected for classical dsDNA structures, demonstrating that they are essentially double-stranded throughout their length (compare Figure 1B with Figures 2–5). The general resistance of the detected intermediates to the ssDNA-specific nuclease S1 provides further evidence for strand-coupled replication. Moreover, the overall pattern of replication intermediates seen in adult flies was essentially the same in other developmental stages (embryos and larvae) and cultured Schneider S2 cells (Figures 6 and 7).

### Partial Strand-uncoupled Replication in the rRNA Locus

Evidence that replication is not fully strand-coupled over the entire length of the *D. melanogaster* mtDNA was obtained from the results of digestion with restriction enzymes cutting within the rRNA gene region, positioned just downstream of the NCR (Figure S1A in File SI). Digestion in that region by Bsp1407I or BshNI did produce a characteristic eyebrow arc (Figure 5A), indicative of a restriction site blocked for cleavage on one (or both) strand(s) [14], [24]. S1 nuclease treatment abolished this eyebrow arc, showing that the region of the blocked site contains ssDNA, and thus indicating single-strandedness in this region as the cause of non-digestion (Figure 5B). Additionally, an arc extending downwards from uncut circles, which would be formed by circular molecules with variable ssDNA regions (i.e. gapped circles, Figure S2 in File SI) can also be seen (ss c in Figure 5). The eyebrow arcs revealed by Bsp1407I or BshNI digestion accounted for only a minor proportion of the replication intermediates, since more conventional dsDNA structures (i.e. dY arcs) were clearly visible as well, in both digests. Since no eyebrow arc was observed in any other digest, we infer that the region of the genome in which synthesis of the two strands is not fully coupled, at least in some molecules, is limited, extending no further than the region upstream of the NdeI site. This partially single-stranded region would also be expected to affect the migration and nuclease sensitivity of bubble intermediates, effectively generating a species of lower molecular weight, less electrophoretically retarded than conventional duplex bubbles, due to the partial ssDNA content. Consistent with this prediction, a shallow sub-bubble arc migrating below the conventional bubble arc was observed (Figures 2–4). This was removed by S1 nuclease treatment, which converted sub-bubbles to linears and/or Y-like structures.

**Figure 8:**
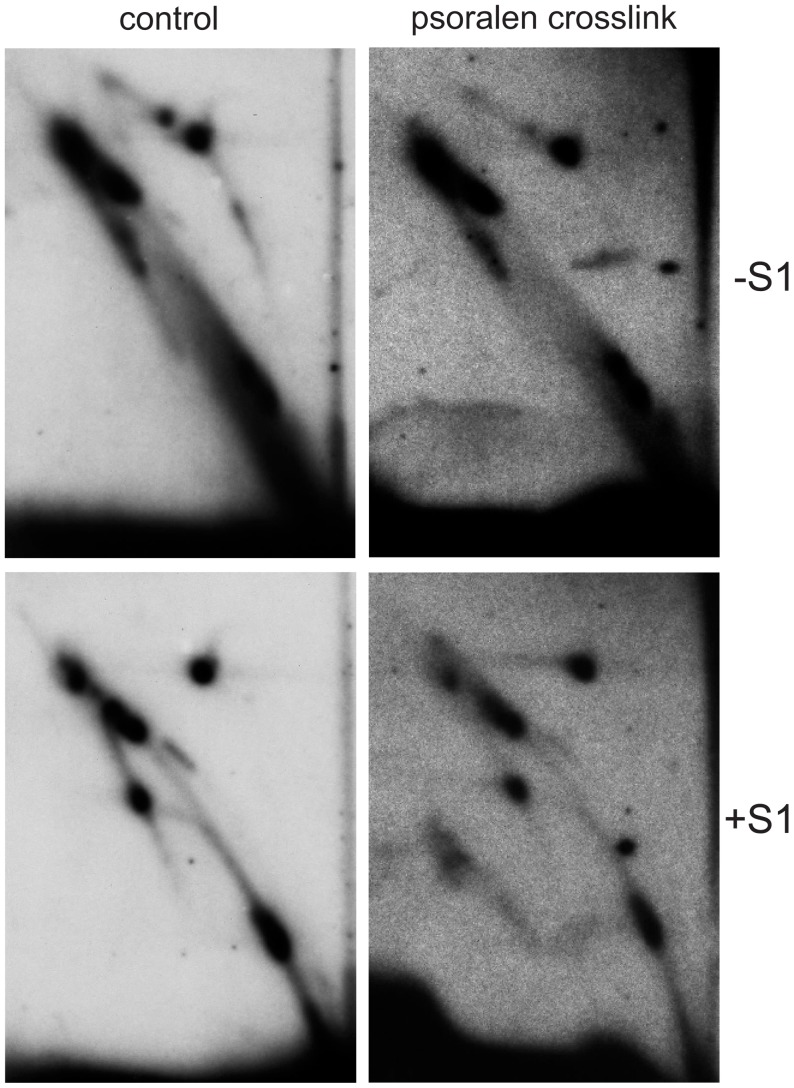
2DNAGE of Bsp1407I-digested mtDNA from S2 cells UV crosslinked in vivo. mtDNA was extracted from psoralen/UV crosslinked cells (right panels) or from control cells (left panels), and visualized with probe 6 after restriction digestion and subsequent S1 nuclease treatment (bottom panels) or no treatment (top panels). The eyebrow arc was still removed by S1 treatment of Bsp1407I-digested intermediates, even in material from crosslinked cells.

RNA has been reported to be present in the lagging strand of replicating vertebrate mtDNA [14]. It was therefore possible that, if RNA were present, its degradation from RNA-DNA heteroduplexes in the course of extraction might artificially produce species that were partially single-stranded in the rRNA gene region. We therefore carried out UV-induced interstrand cross-linking in live Schneider S2 cells treated with psoralen. Psoralen/UV covalently links pyrimidines in opposite strands of duplex nucleic acids. Thus, it should protect an otherwise labile RNA-DNA heteroduplex from degradation. After prior cross-linking, the eyebrow structure was still removed by S1 treatment (Figure 8), consistent with the region of single-strandedness being present *in vivo*. The same S1-sensitive eyebrow arc was seen in Bsp1407I or BshNI digests of material from adult flies, S2 cells, embryos and larvae (Fig. 5–7), indicating that is applies generally in *D. melanogaster.* The presumed structures of the RIs giving rise to the eyebrow, gapped circle and sub-bubble arcs is depicted in Figure S2, in File SI, along with the forms predicted to be generated from them by S1 nuclease treatment.

### Replication Pauses in Distinct Regions

Rather than displaying homogeneous arcs, the pattern of mtDNA replication intermediates from *D. melanogaster* showed several strong signal concentrations mapping to specific regions of the mitochondrial genome, indicative of replication pausing or slow zones [25], [26], and characteristic of unidirectional replication (see legend to Fig. 2). Two such slow zones were located either on the bubble or dY arc, depending on the site of restriction digestion. In the MbiI, XhoI and StyI digests, both slow zones were located on the bubble arc (Fig. 2&3). In the PstI digest, one of the slow zones was seen on the bubble arc, the other on the dY arc (Fig. 3). In the case of Bsp1407I and BshNI digests (Fig. 5), both of the major pause regions appeared on the dY arc. These different behaviors allow the slow zones to be mapped approximately to regions downstream of the NdeI and StyI sites (Figure 1), respectively. The signal from the former slow zone (sz1) is split by NdeI digestion (Fig. 4) into portions on both the bubble and dY arcs. The latter slow zone (sz2) is followed by a long but less concentrated region of accumulated RIs extending to the edge of the NCR. Note that, due to gel compression, this region is not separable from slow zone sz2 in the NdeI, BshNI and Bsp1407I digests.

Another conspicuously strong intermediate type was present on the X-arc in all of the digests, representing either products of recombination or pre-termination forms, their migration depending on the position of the cruciform junction (t in Fig. 2–5). Structures with a cruciform junction at the center of a given fragment are maximally retarded in the second dimension, and are thus found at the apex of the X-arc, whereas those with a cruciform junction displaced towards one end of the fragment migrate closer to the base of the X-arc [7], [9]. The position of strongest signal on the X-arc was close to its apex in the PstI and StyI digests, rather lower in other digests, and closest to the base in the MbiI digest (Fig. 2&3), indicating that cruciform junctions are mainly located towards the middle of the NCR, i.e. coincident with the origin/terminus.

An additional RI (t’ in Fig. 2&5), of lower molecular weight than the previously described X structures, and which was relatively more sensitive to S1 nuclease than the final termination intermediate (t), was seen in most digests. This was most clearly visible when the major junctional region was close to the end of the fragment. Delayed completion of the lagging strand in the rRNA gene region, which also gives rise to the eyebrow and sub-bubble arcs, is predicted to create exactly such a species (Figure S2 in File SI). S1 nuclease digestion in this region would releases one of the arms, thus converting the t’ to a Y-structure (close to the apex of the Y-arc in the NdeI digest in Fig. 4), as seen.

## Discussion

Our data provide evidence for unidirectional replication in *D. melanogaster* starting within the NCR and proceeding in the direction of the rRNA locus. Almost all intermediates that were detected by 2D electrophoresis were found to be fully double-stranded. This can only occur if both strands are being synthesized in tandem at the replication fork, thus implying conventional, strand-coupled DNA synthesis as the prevalent mode of mtDNA replication in *D. melanogaster*.

Our findings indicate that the previously proposed strand-displacement model for insect mtDNA replication needs to be revised. Previously published TEM analyses of animal mtDNA replication intermediates (RIs) were interpreted in support of the strand-displacement model, whereby the synthesis of the second strand is initiated after the replication fork has traversed 2/3 (mammals) or >90% (*D. melanogaster*) of the mitochondrial genome length. However, such strand-asynchronous replication would give rise to molecular species with extensive ssDNA regions. These would be very poor substrates for restriction endonucleases and, being of significantly lower molecular mass, would migrate along different trajectories on 2DNAGE than dsDNA intermediates. They would also be highly sensitive to S1 nuclease treatment, which would dramatically affect the detection and mobility of such species. None of these effects was observed with a series of endonucleases, each cutting once in the *D. melanogaster* mitochondrial genome, and encompassing the entire coding region. The major species that were detected instead displayed characteristics of classical, strand-synchronous replication products.

The principal replication intermediates revealed in the study were qualitatively similar, irrespective of the biological source, i.e. adult flies, larvae, embryos and cultured cells of *D. melanogaster*. The differences between our conclusions and those of earlier studies are therefore not attributable to developmental changes in the mode of mtDNA replication. Instead, we would argue that, as in mammals [16], the different methods of sample preparation for TEM and for 2DNAGE must have a material bearing on the types of intermediate that can be detected.

Our findings corroborate the earlier results obtained by TEM, in the sense that, using an independent method, we find evidence for the same two classes of theta-replication intermediate, namely those that are fully duplex throughout their length, and a subset, which we find to be the minority, which are partially single-stranded on one branch. One of the previous TEM analyses, using mitochondrial preparations from embryos, with subsequent fractionation by CsCl density-gradient centrifugation, detected such a mixed population of fully duplex (theta) and partially single-stranded RIs, distinguished on the basis of their sensitivity to restriction endonuclease digestion [5]. The earlier report, using similar methods and source material, described only theta-type RIs [6]. The latter publication also reported a low rate of mtDNA replication in embryos compared to cultured cells, an observation confirmed by our 2DNAGE results, which revealed a lower relative abundance of replicating molecules in embryos compared to other sources (compare Figure 6 to Figure 1–5).

Nevertheless, our results differ from those of previous TEM studies, by indicating a more restricted region of single-strandedness, which was mapped to a specific region of the genome, namely the rRNA genes. The RIs suggested to be of the strand-asynchronous type, based on the earlier TEM data, were derived from single bands visualized on preparative CsCl/ethidium bromide density-gradients, whilst theta-structures originated from the region between mtDNA circles and high molecular weight nuclear DNA [5], [6]. These different procedures would have led to the unintended selection or counter-selection of certain topological forms of mtDNA. Finally, sample preparation for TEM may have resulted in partial degradation or other modification of mtDNA replication intermediates, leading to the appearance of aberrant species not present *in vivo*, as earlier inferred for similar studies of mammalian mtDNA replication [15]. In addition, it should be noted the earlier TEM analysis that led to the inference of strand-displacement replication actually included partial denaturation as an explicit step in sample preparation [5]. Nicks in nascent strands behind an advancing replication fork might have caused a stepwise removal of newly synthesized daughter strand under partially denaturing conditions. In fact, replicating molecules with single-stranded segments at both the origin and fork, but with fully duplex dsDNA in between, were indeed reported [5]. Together these factors can account for the depletion of the fully double-stranded intermediates that our study reveals as the prevalent class. In contrast, our mtDNA was isolated from a highly pure, sucrose density gradient-derived mitochondrial fraction free of other cellular constituents or added nucleases, without any selection between topological forms or additional treatments prior to endonuclease digestion.

The limited stretch of ssDNA encompassing the rRNA locus was observed only in a subset of replicating molecules, in the present study. This region retained its partially single-stranded nature even in some molecules that had completed almost a full round of synthesis, as inferred from their limited sensitivity to S1 nuclease. We confirmed that the presence of this single-stranded region was not the result of degradation of the RNA strand of a region of RNA/DNA hybrid, by means of UV-induced interstrand crosslinking with psoralen. This method should preserve any such duplex, rendering the RNA component insensitive to degradation by RNases and, therefore, the whole region resistant to S1 nuclease. The effectiveness of the crosslinking reaction was verified by the expected decrease in hybridization sensitivity (not shown), resulting from covalent bonds formed between DNA strands that interfere with probe hybridization. However, the species containing ssDNA, which were revealed as eyebrow arcs, remained fully sensitive to S1 nuclease after treatment with psoralen and UV light. Whilst we cannot exclude transient or limited RNA/DNA heteroduplexes forming during replication in other regions of the genome, the persistent sensitivity of the rRNA region to S1 nuclease serves as an internal control in our experiments, validating the conclusion that the remainder of the molecule during replication is essentially double-stranded.

**Figure 9:**
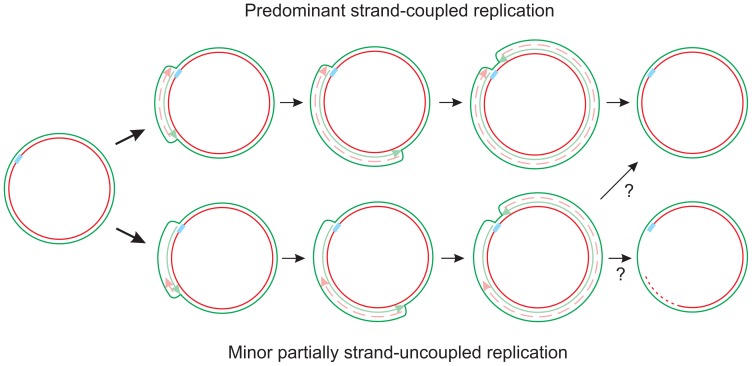
Proposed mode of mtDNA replication in *D. melanogaster*. Diagrams proposing two coexisting *D. melanogaster* mtDNA replication modes. Diagrams in top line indicate conventional, strand-coupled replication. Those in the bottom line denote molecules in which the lagging-strand initiates only after the replication fork has traversed the rRNA gene region, and is not completed until a late stage in replication, leaving a single-stranded gap (possibly shorter than shown) to be filled in. Blue box marks the origin of replication, elongating strands are shown in light green and pink (lagging strand as a dashed line). The region between NdeI and Bsp1407I restriction sites, where the provisional lagging-strand terminates in an incompletely replicated mtDNA molecule, is shown as a red dashed line.

The data we report here enables us to elaborate a tentative model for mtDNA replication in *D. melanogaster* (Figure 9). In this model, most molecules are replicated by a conventional Cairns’ type of replication, with concomitant synthesis of both strands, initiating unidirectionally within the NCR by an as-yet unknown mechanism. In a minority of molecules, lagging-strand synthesis initiates only after the rRNA region has been traversed, leaving a zone of single-strandedness near the origin that is not filled in until late in the replication cycle, as evidenced by the X-like termination intermediates that still retain this region of S1-sensitivity. This mode of replication has many similarities with that commonly exhibited by bacterial plasmids [9].

The significance of a sub-population of mtDNA molecules that retain a single-stranded region, and thus have not completed replication, is unclear. They could be considered in some ways analogous to mammalian mtDNA molecules containing a D-loop, even though in this latter case the triplex D-loop encompasses only the NCR. The D-loop is widely believed to perform some regulatory role in mitochondrial homeostasis, although its exact function remains unknown, despite many years of research. Alternatively, the fact that single-strandedness maps to the rRNA genes suggests that it may simply be a consequence of delayed lagging-strand initiation, in a region that is heavily transcribed in the opposing direction.

The observation of replication pause regions or slow zones is intriguing. In principle, it might reflect any of several phenomena, based on previous findings, including abnormal base composition [27], [28], specific structures such as triplexes [29] or inverted repeats [30], protein occupancy [31]–[33] or local variation in transcriptional activity [34]. Determining the exact physiological significance of pausing in *Drosophila* mtDNA is obviously an important goal of future research.

The elucidation of the basic mechanism of mtDNA replication in *Drosophila*, and the finding that it mirrors what has been found previously in vertebrates, should now enable this key model organism to be exploited, in order to learn more about the machinery of mtDNA maintenance. The panoply of molecular tools available to *Drosophila* researchers thus opens up the key issues to analysis, using a tractable genetic system.

## Materials and Methods

### Flies, Cell-lines and Culture

Schneider’s S2 cell-line [35] was cultured in Schneider’s Medium (Sigma-Aldrich), including 10% fetal bovine serum, at 25°C, usually with the addition of 100 U/ml penicillin/streptomycin (Lonza). Cells were passaged every 3–4 d at a density of 0.5×10^6^ cells/ml. Standard *D. melanogaster* strain Oregon R (wild-type) was cultured as described previously [36].

### DNA Isolation for 2DNAGE

For 2DNAGE, mitochondrial DNA was extracted from flies collected at different developmental stages either by anesthetizing with CO_2_ (flies, between 500–1500 per prep) or from food plates attached to mating chambers (larvae and embryos, between 1000–2000 per prep). In the case of larval samples food debris was removed by repeated washing with water, followed by washing with ice-cold homogenization buffer (HB, 225 mM mannitol, 75 mM sucrose, 10 mM Tris-HCl, 1 mM EDTA, 0.1% BSA pH 7.6). All subsequent steps were carried out at 4°C, except where stated. The collected flies, larvae or embryos were Dounce-homogenized in 5–7 ml of HB by 10 strokes of a loose-fitting pestle, and the homogenate transferred to a 15 ml Falcon tube. Residual material was rehomogenized in a further 5–7 ml of HB, with 1–2 further strokes, and added to the first homogenate. Body parts and other debris were removed by 3 rounds of centrifugation at 1,000 *g_max_*. Crude mitochondria were then pelleted by centrifugation for 10 min at 9,000 *g_max_*, followed by washing with 15 ml of HB and re-centrifugation. The pellet was resuspended in 500 µl of HB, overlaid onto a sucrose step-gradient (1/1.5 M in 20 mM HEPES-KOH, pH 7.2), and centrifuged for 1 h at 180,000 *g_max_*. The mitochondrial fraction was collected from the interface between two sucrose steps, diluted with at least 1 volume of HB and recovered by centrifugation for 5 min at 12,000 *g_max_*. The pellet was resuspended in 1 ml of nucleic acid isolation solution (75 mM NaCl, 25 mM EDTA, pH 8.0), after which 200 µl of 10% SDS and 200 µg of Proteinase K were added. The reaction was incubated at room temperature for 30 min, followed by extraction with phenol-chloroform-isoamyl alcohol mixture (25∶24∶1). Nucleic acids were precipitated from the aqueous phase using 0.2 vol 10 M ammonium acetate and 2 vol of ethanol at −20°C overnight, followed by centrifugation for 16,000 *g_max_* for 20 min and washing with 70% ethanol. For DNA extraction from S2 cells, confluent cells were harvested from between one and three 175 cm^2^ flasks, washed once with medium, then with ice-cold PBS, with recovery in each case by centrifugation for 3 min at 600 *g_max_*. The cells were then resuspended in 2 ml of 0.1 x cell homogenization buffer (CHB, 40 mM Tris-HCl, 25 mM NaCl, 50 mM MgCl_2_, pH 7.8) and incubated on ice for 6 min (at 4°C, as for all subsequent steps in the procedure). The cell suspension was lysed in a Dounce homogenizer by 10–15 strokes of a tight-fitting pestle, after which 200 µl of 10 x CHB was added. The homogenate was centrifuged for 3 min at 1,200 *g_max_*. The supernatant was transferred to a fresh tube and centrifugation repeated. Crude mitochondria were pelleted in a fresh tube at 14,000 *g_max_* for 3 min, resuspended in 500 µl of HB, then processed by sucrose density-gradient centrifugation and nucleic acid extraction as for the material from flies.

### Psoralen/UV Crosslinking of Mitochondrial Nucleic Acids *in *vivo

S2 cells (1–2 confluent 175 cm^2^ flasks) were collected by centrifugation at 600* g_max_* for 3 min at room temperature, washed once with PBS, then resuspended in 2 ml of PBS containing 2.5 µM 4,5′,8 - trimethylpsoralen (trioxsalen, Sigma-Aldrich) and incubated on ice in the dark for 5 min. Cells were then irradiated with UV-A (200 mJ/cm^2^) and collected by centrifugation as above. After washing once with PBS, mitochondrial and nucleic acid isolation was carried out using the standard procedure.

### 2DNAGE and Southern Blot-hybridization

2DNAGE used TBE buffer in both dimensions, with typically 5–10 µg of total nucleic acid per track. Restriction endonuclease (Fermentas) reactions were incubated at 37°C for 4 h with 4 units of enzyme per µg of mitochondrial nucleic acid, in manufacturer’s recommended buffers. Any subsequent treatment with S1 nuclease (Fermentas) was also carried out according to the enzyme manufacturer’s protocol, after stopping the initial restriction digest and removing enzyme by phenol/chloroform extraction and ethanol precipitation. S1 nuclease digestion (0.2 u per µg of nucleic acid) was for 2 min at room temperature. The control (S1 untreated) sample was subject to the same incubation conditions with identical buffers, in order to rule out possible changes in RIs due purely to the conditions. First dimension electrophoresis in 0.28% agarose was run without ethidium bromide at 1.7 V/cm at room temperature for 24 h. After the first dimension the gel was stained with ethidium bromide (300 ng/ml in TBE) and documented. Individual sample lanes were cut and placed into the second-dimension tray, rotated through 90°. The second-dimension 0.58% agarose gel containing 300 ng/ml ethidium bromide was cast around it at 55°C, and electrophoresis was conducted with constant buffer circulation at 1.8 V/cm at room temperature for 67 h. After electrophoresis, second-dimension gels were processed for Southern blotting by successive washes (20 min each) in 0.25 M HCl, twice in 0.5 M NaOH, 1.5 M NaCl and once in 2 M NaCl, 1 M Tris-HCl pH 7.2. Nucleic acids were transferred to a nylon membrane (Amersham Hybond) by overnight capillary transfer in 2 M NaCl, 1 M Tris-HCl pH 7.2, after which membranes were rinsed in 6 x SSC, cross-linked with UV-light and stored in 6 x SSC until hybridization. Radioactive probes were generated by PCR using 100 ng of a gel-purified PCR product generated from *Drosophila* mtDNA as a template but substituting 0.8 µM [γ-^32^P]-dCTP (Perkin-Elmer, 3000 Ci/mmol) for unlabeled dCTP in the reaction mix, which contained 0.2 mM each of the other 3 unlabeled deoxynucleotides, and primers (10 pmol per reaction). Primers for probe 3 were Dm3276F (5′-AACTATTTTACCAGCAATTATTTTACT-3′) and Dm3840R (5′-CAGTCATCTAATGAAGAGTTATTTCTA-3′), and for probe 6 primers Dm6801F (5′-AAATCAATCAATTTAATATTCTACCTC-3′) and Dm7378R (5′-ATTAACAATATTTATAGCTGGATTAGG-3′). Both for template and probe synthesis, PCR conditions were as follows: initial denaturation at 95°C for 4 min, followed by 29 cycles of 95°C for 30 s, 50°C for 30 s, 72°C for 1 min, with final extension at 72°C for 5 min. Probes were purified by gel-filtration on Sephadex G-50 spin-columns (Roche). Blot-hybridization was carried out overnight at 55°C in hybridization buffer (250 mM sodium phosphate, 7% SDS, 1 mM EDTA, pH 7.2) preceded by a 1 h pre-hybridization in the same buffer, also at 55°C. After hybridization the membrane was washed once for 20 min with 5 x SSC, 0.1% SDS, and twice for 20 min with 1x SSC, 0.1% SDS, then exposed at –70°C to autoradiography film (Kodak BioMax MS or Fuji Super RX film depending on signal strength) for between 1 h and 14 d, to generate the range of exposures shown in the figures.

### Gel Image Processing

Gel images shown in the figures are optimal exposures revealing the different species of RI seen. Scanned images were cropped for clarity and optimized, in some cases, for brightness and contrast, but no gamma corrections or any other manipulations were carried out, apart from the addition of annotations as requested by reviewers of the manuscript.

## Supporting Information

File SI **This contains Figures S1, S2 and their individual legends.**
(PDF)
