# Supplementary material for: Analysis of Replication Intermediates Indicates That Drosophila melanogaster Mitochondrial DNA Replicates by a Strand-Coupled Theta Mechanism
Source: PLoS One. 2013 Jan 4;8(1):e53249. doi: 10.1371/journal.pone.0053249 (PMC3537619; doi:10.1371/journal.pone.0053249)
Supplement: File SI — This contains Figures S1, S2 and their individual legends. (PDF) [file pone.0053249.s001.pdf]

SUPPLEMENTARY DATA FILE

Legend to Supplementary Figures

Supplementary Figure S1. Position of structures relevant to mtDNA replication and RI diagrams.

This figure provides more detail to that shown in Figure 1, omitted from the main figure for clarity. A. Supplementary diagram of *D. melanogaster* mt genome with positions of probes, restriction enzyme sites and regions relevant to replication as explained in Results. sz1 and sz2 – replication slow zones 1 and 2. B. Supplementary diagram of main RI migration paths with the different arcs (and corersponding labels) shown in different colors: uc – uncut circles, b – bubble arc, sub-b – sub-bubble arc, Y – Y or Y-like arc, resulting from strand-breakage at the origin or fork, dY – double-Y arc, X – cruciform structures, ey – eyebrow arc. Bold black line shows the area where Y-like and dY species are not resolved, due to the liimitations of the gel system. C. Depiction of species derived from digestion of RIs with different restriction enzymes used in the study. Replication fork progression from the origin is, in all cases to the right, as shown, with pausing, as indicated, in sz1, sz2 (grey boxes) and t (termination zone). For BshNI and Bsp1407I digests there are two classes of RIs revealed: fully duplex theta (Cairns-type) forms and partially single-stranded species (which give rise to an eyebrow arc). X marks the position of the blocked restriction site on the single-stranded branch (dashed line).

Supplementary Figure S2. Proposed structures of replicating molecules containing singe-stranded regions

Diagrams show the partially single-stranded structures expected due to suppressed initiation of lagging-strand synthesis within the rRNA gene region and the consequences of treating those structures with S1 nuclease, following restriction enzyme digestion. A) Expected migration of partially ssDNA RIs on 2DNAGE before (blue) and after S1 treatment (red/pink). Eyebrow (ey), double-Y (dY), X and sub-bubble (sub-b) structures containing single-stranded segments are converted by S1 to linear or Y-like structures migrating either on the standard Y-arc (red line) or in the sub-Y area (shown in pink). Gapped circles (ss c) are converted to sub-genomic linear fragments. B) Diagrams show partially single-stranded RIs before and after S1 treatment, following digestion either with XhoI, BshNI or Bsp1407I. dsDNA is shown as a continuous line and ssDNA segments resulting from delayed lagging-strand synthesis as a dashed line. Depending on the extent of progression of the fork and the position of the restriction site, different Y-like structures are produced that will migrate on 2DNAGE gels in different regions of the standard Y arc (shown in red in panel A) and the sub-Y region (shown in pink in panel A). Following segregation of daughter circles, molecules in which lagging-strand synthesis in the rRNA locus has not yet occurred remain undigestible with BshNI or Bsp1407I, and migrate as gapped circles. S1 nuclease converts them to linear fragments of sub-genomic length. Grey boxes are slow zones and t marks the position of termination.

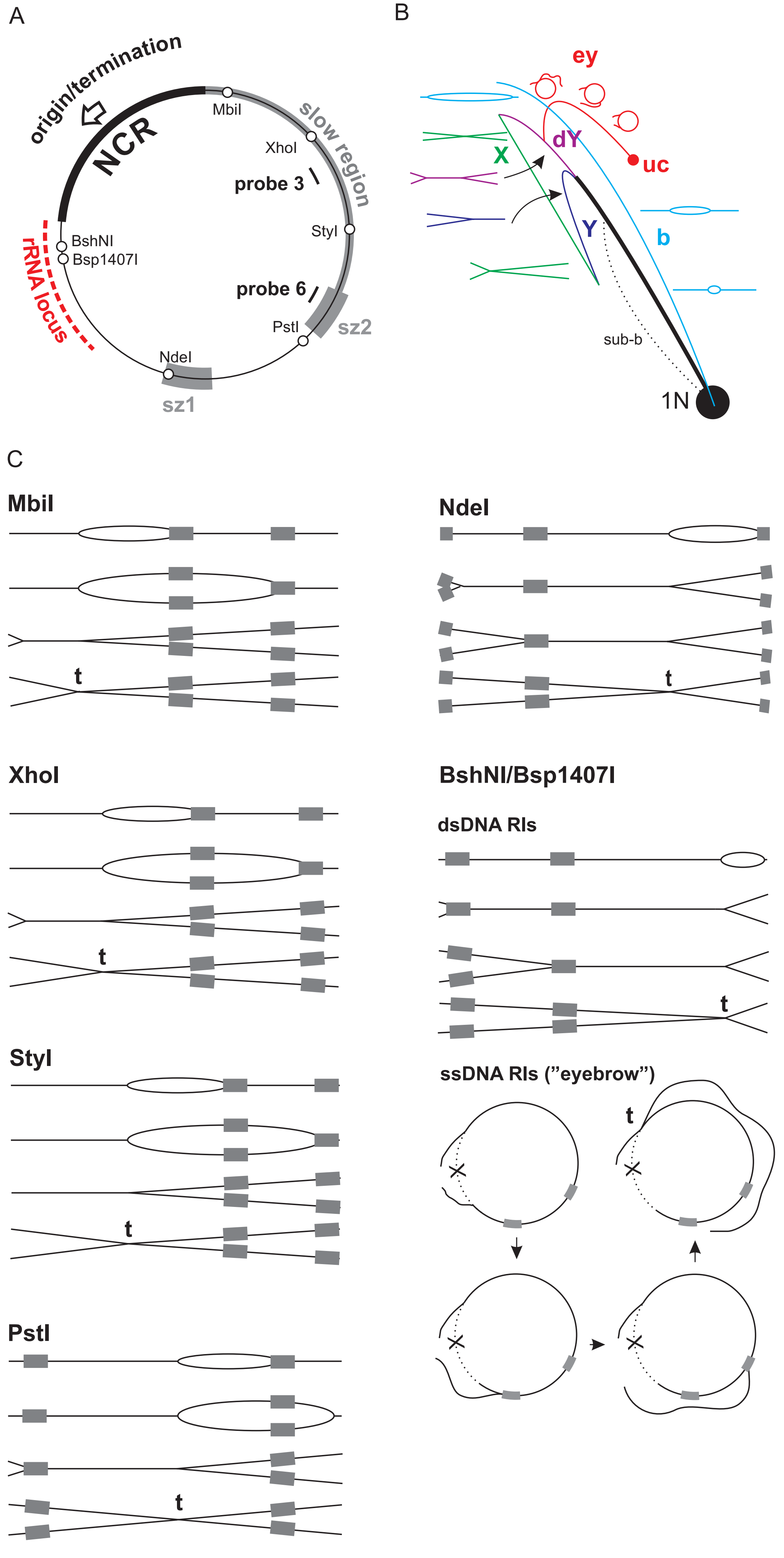
A
origin/termination
NCR
MbiI
XhoI
slow region
probe 3
StyI
probe 6
sz2
PstI
NdeI
sz1
BshNI
Bsp1407I
rRNA locus
B
ey
dY
uc
X
Y
b
sub-b
1N
C
MbiI
t
NdeI
t
XhoI
t
BshNI/Bsp1407I
dsDNA RIs
t
StyI
t
ssDNA RIs ("eyebrow")
t
PstI
t

Figure S1

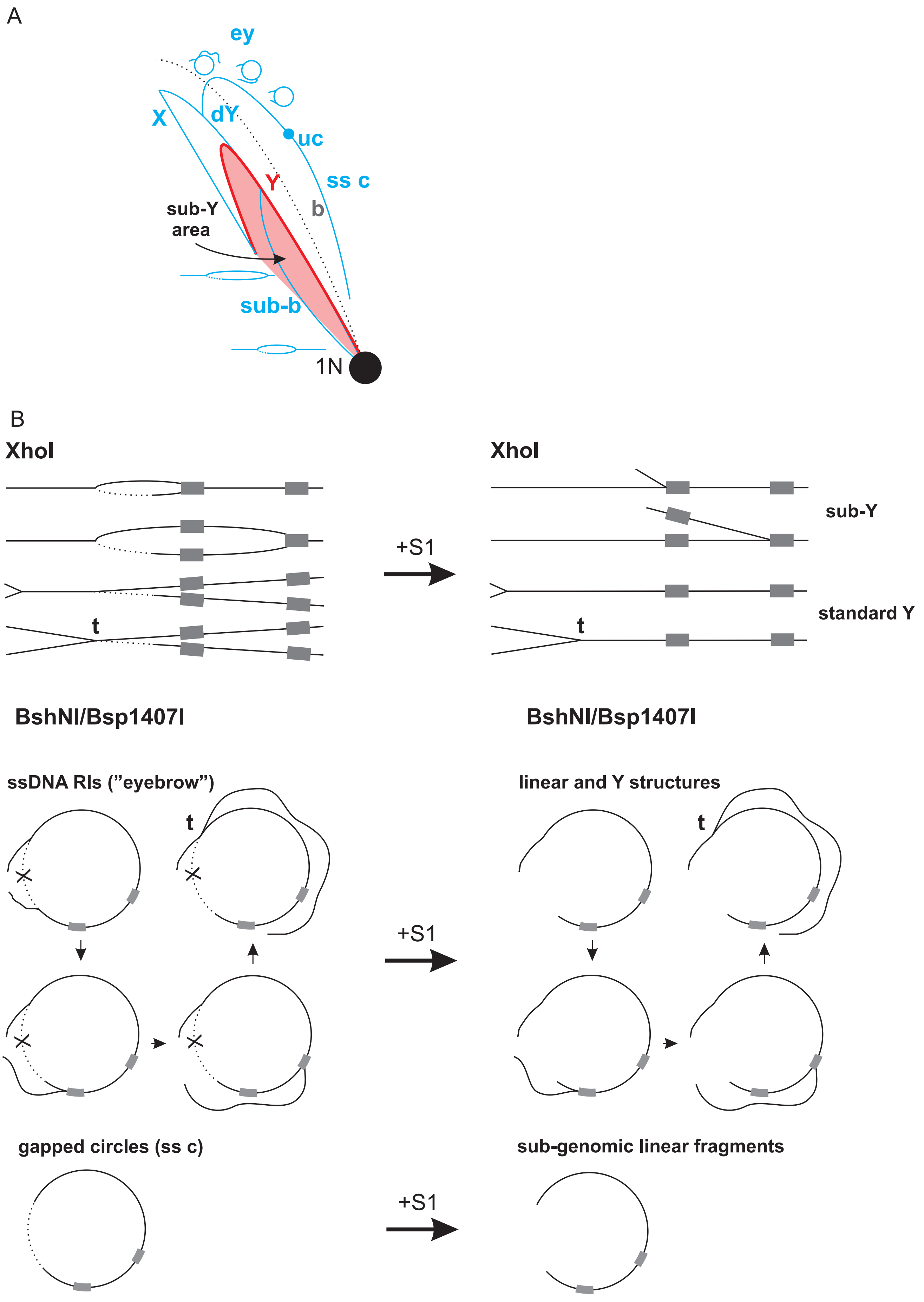
A
ey
X
dY
uc
ss c
Y
b
sub-Y
area
sub-b
1N
B
XhoI
+S1
XhoI
sub-Y
t
standard Y
t
BshNI/Bsp1407I
BshNI/Bsp1407I
ssDNA RIs (”eyebrow”)
t
+S1
linear and Y structures
t
gapped circles (ss c)
+S1
sub-genomic linear fragments

Figure S2
